# 4-Eth­oxy­carbonyl-*N*,*N*,*N*-trimethyl­anilinium iodide

**DOI:** 10.1107/S1600536811049361

**Published:** 2011-11-30

**Authors:** Xiao-Yan Tang

**Affiliations:** a College of Chemistry & Materials Engineering, Jiangsu Laboratory of Advanced Functional Materials, Changshu Institute of Technology, Changshu, 215500 Jiangsu, People’s Republic of China

## Abstract

In the title molecular salt, C_12_H_18_NO_2_^+^·I^−^, the C atoms of the ethyl group are disordered over two sets of sites [occupancies of 0.76 (4) and 0.24 (4)]. In the crystal, ion pairs linked by weak C—H⋯I interactions occur.

## Related literature

The title compound is a key intermediate in the preparation of carboxylates. A wide variety of model metal carboxylic compounds has been prepared with the aim of mimicing the structures and functions of the active sites of metal metalloenzymes, see: Liu *et al.* (2004 ▶).
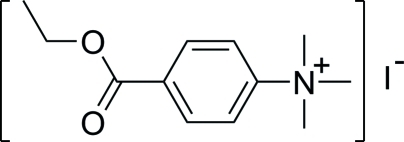


## Experimental

### 

#### Crystal data

C_12_H_18_NO_2_^+^·I^−^
*M* *_r_* = 335.17
Triclinic, 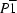

*a* = 7.4790 (15) Å
*b* = 10.008 (2) Å
*c* = 10.158 (2) Å
α = 71.16 (3)°
β = 83.30 (3)°
γ = 84.62 (3)°
*V* = 713.4 (2) Å^3^
*Z* = 2
Mo *K*α radiation
μ = 2.23 mm^−1^
*T* = 293 K
0.3 × 0.2 × 0.2 mm

#### Data collection

Rigaku SCXmini diffractometer
Absorption correction: multi-scan (*REQAB*; Jacobson, 1998 ▶) *T*_min_ = 0.594, *T*_max_ = 0.644
7462 measured reflections
3258 independent reflections
2708 reflections with *I* > 2σ(*I*)
*R*_int_ = 0.032

#### Refinement

*R*[*F* ^2^ > 2σ(*F* ^2^)] = 0.044
*wR*(*F* ^2^) = 0.082
*S* = 1.14
3258 reflections
169 parameters
38 restraints
H-atom parameters constrained
Δρ_max_ = 0.32 e Å^−3^
Δρ_min_ = −0.37 e Å^−3^

### 

Data collection: *CrystalClear* (Rigaku, 2005 ▶); cell refinement: *CrystalClear*; data reduction: *CrystalStructure* (Rigaku/MSC, 2004 ▶); program(s) used to solve structure: *SHELXS97* (Sheldrick, 2008 ▶); program(s) used to refine structure: *SHELXL97* (Sheldrick, 2008 ▶); molecular graphics: *SHELXTL/PC* (Sheldrick, 2008 ▶) and *ORTEPII* (Johnson, 1976 ▶); software used to prepare material for publication: *SHELXTL/PC* and *PLATON* (Spek, 2009 ▶).

## Supplementary Material

Crystal structure: contains datablock(s) I, global. DOI: 10.1107/S1600536811049361/bx2381sup1.cif

Structure factors: contains datablock(s) I. DOI: 10.1107/S1600536811049361/bx2381Isup2.hkl

Supplementary material file. DOI: 10.1107/S1600536811049361/bx2381Isup3.cml

Additional supplementary materials:  crystallographic information; 3D view; checkCIF report

## Figures and Tables

**Table 1:** Hydrogen-bond geometry (Å, °)

*D*—H⋯*A*	*D*—H	H⋯*A*	*D*⋯*A*	*D*—H⋯*A*
C5—H5⋯I1^i^	0.93	3.02	3.932 (4)	166

Symmetry code: (i) 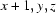.

**Fig. 1.**
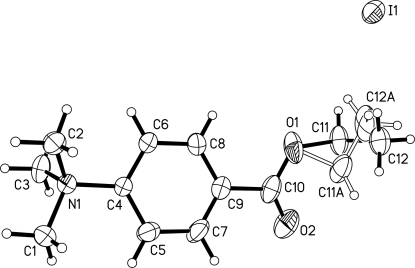
ORTEP-II (Johnson, 1976) plot of complex (I) at the 30% probability level. Hydrogen atoms are drawn as spheres of arbitrary radii. The C11 and C12 atoms of ethyl group are disorder over two sites.

## supplementary crystallographic information

### Comment

Recently, the chemistry of metal complexes of carboxylates has been receiving an increasing attention. To date, a wide variety of model metal carboxylic compounds has been prepared with the aim to mimic the structures and functions of the active sites of metal metalloenzymes [Liu *et al.*, 2004]. The title compound (I), is a key intermediate in the preparation of carboxylates, which we are designing for the synthesis of metal complexes. The structure of the title compound, [C_12_H_18_NO_2_]^+ ^.I^-^, comprises discrete ions which are interconected by weak C—H···I hydrogen bonds. These hydrogen bonds appear to complement the Coulombic interaction and help to stabilize the structure further.The molecular structure is stabilize by one intramolecular C—H···O hydrogen bond. The C atoms of ethyl group are disorder over two occupied positions [0.76 (4)/0.24 (4)].

### Experimental

The title compound was synthesized by reaction of 4-Dimethylamino-benzoic acid ethyl ester (0.966 g, 5 mmol) and Iodomethane (0.710 g, 5 mmol) in acetone (40 ml). The solution was vigorously stirring for 24 h to afford white precipitates. The precipitates were collected by filtration, re-dissolved in MeOH (10 ml) then allowed to stand for several days to produce white crystals (I). Yield: 1.44 g (86%). The crystal used for the crystal structure determination was obtained directly from the above preparation. Analysis, found: C, 43.32; H, 5.31; N, 4.12%. calculated. for C_12_H_18_INO_2_: C, 43.00; H, 5.41; N, 4.18%.

### Refinement

Carbon-bond H atoms were positioned geometrically (C—H = 0.97 Å for methylene group, C—H = 0.96 Å for methyl group, C—H = 0.93 Å for phenyl group), and were included in the refinement in the riding mode approximation, with *U*_iso_(H) = 1.2*U*_eq_(C) for methylene group and phenyl group and *U*_iso_(H) = 1.5*U*_eq_(C) for methyl group. The ethyl group C atoms are disorder over two occupied positions [0.76 (4)/0.24 (4)].

### Crystal data

**Table:**

C_12_H_18_NO_2_^+^·I^−^	*Z* = 2
*M**_r_* = 335.17	*F*(000) = 332
Triclinic, *P*1	*D*_x_ = 1.560 Mg m^−^^3^
Hall symbol: -P 1	Mo *K*α radiation, λ = 0.71073 Å
*a* = 7.4790 (15) Å	Cell parameters from 7462 reflections
*b* = 10.008 (2) Å	θ = 3.7–27.5°
*c* = 10.158 (2) Å	µ = 2.23 mm^−^^1^
α = 71.16 (3)°	*T* = 293 K
β = 83.30 (3)°	Block, colourless
γ = 84.62 (3)°	0.3 × 0.2 × 0.2 mm
*V* = 713.4 (2) Å^3^	

### Data collection

**Table:**

Rigaku SCXmini diffractometer	3258 independent reflections
Radiation source: fine-focus sealed tube	2708 reflections with *I* > 2σ(*I*)
graphite	*R*_int_ = 0.032
ω scans	θ_max_ = 27.5°, θ_min_ = 3.3°
Absorption correction: multi-scan (*REQAB*; Jacobson, 1998)	*h* = −9→9
*T*_min_ = 0.594, *T*_max_ = 0.644	*k* = −12→12
7462 measured reflections	*l* = −12→13

### Refinement

**Table:**

Refinement on *F*^2^	Primary atom site location: structure-invariant direct methods
Least-squares matrix: full	Secondary atom site location: difference Fourier map
*R*[*F*^2^ > 2σ(*F*^2^)] = 0.044	Hydrogen site location: inferred from neighbouring sites
*wR*(*F*^2^) = 0.082	H-atom parameters constrained
*S* = 1.14	*w* = 1/[σ^2^(*F*_o_^2^) + (0.025*P*)^2^ + 0.3117*P*] where *P* = (*F*_o_^2^ + 2*F*_c_^2^)/3
3258 reflections	(Δ/σ)_max_ = 0.006
169 parameters	Δρ_max_ = 0.32 e Å^−^^3^
38 restraints	Δρ_min_ = −0.37 e Å^−^^3^

### Special details

**Table:**

Geometry. All e.s.d.'s (except the e.s.d. in the dihedral angle between two l.s. planes) are estimated using the full covariance matrix. The cell e.s.d.'s are taken into account individually in the estimation of e.s.d.'s in distances, angles and torsion angles; correlations between e.s.d.'s in cell parameters are only used when they are defined by crystal symmetry. An approximate (isotropic) treatment of cell e.s.d.'s is used for estimating e.s.d.'s involving l.s. planes.
Refinement. Refinement of *F*^2^ against ALL reflections. The weighted *R*-factor *wR* and goodness of fit *S* are based on *F*^2^, conventional *R*-factors *R* are based on *F*, with *F* set to zero for negative *F*^2^. The threshold expression of *F*^2^ > σ(*F*^2^) is used only for calculating *R*-factors(gt) *etc*. and is not relevant to the choice of reflections for refinement. *R*-factors based on *F*^2^ are statistically about twice as large as those based on *F*, and *R*- factors based on ALL data will be even larger.

### Fractional atomic coordinates and isotropic or equivalent isotropic displacement parameters (Å^2^)

**Table:**

	*x*	*y*	*z*	*U*_iso_*/*U*_eq_	Occ. (<1)
I1	0.20239 (3)	0.72149 (3)	0.14771 (3)	0.06381 (12)	
N1	1.2692 (4)	0.2438 (3)	0.1056 (3)	0.0486 (7)	
O1	0.5587 (4)	0.3017 (4)	0.5043 (3)	0.0916 (11)	
O2	0.6021 (5)	0.0665 (4)	0.5856 (4)	0.1038 (12)	
C7	1.3112 (6)	0.1205 (5)	0.0509 (5)	0.0749 (13)	
H7A	1.3263	0.0352	0.1276	0.112*	
H7B	1.4203	0.1350	−0.0106	0.112*	
H7C	1.2137	0.1126	0.0007	0.112*	
C9	1.2508 (6)	0.3751 (4)	−0.0189 (4)	0.0627 (10)	
H9A	1.1514	0.3682	−0.0678	0.094*	
H9B	1.3598	0.3842	−0.0804	0.094*	
H9C	1.2294	0.4566	0.0123	0.094*	
C8	1.4246 (5)	0.2575 (5)	0.1829 (5)	0.0694 (12)	
H8A	1.4024	0.3406	0.2114	0.104*	
H8B	1.5344	0.2649	0.1225	0.104*	
H8C	1.4352	0.1756	0.2638	0.104*	
C4	1.1010 (4)	0.2256 (4)	0.2045 (4)	0.0462 (8)	
C3	1.0206 (6)	0.0988 (4)	0.2559 (5)	0.0686 (12)	
H3	1.0645	0.0225	0.2258	0.082*	
C5	1.0316 (5)	0.3386 (4)	0.2463 (5)	0.0661 (12)	
H5	1.0852	0.4248	0.2101	0.079*	
C2	0.8712 (6)	0.0872 (5)	0.3544 (5)	0.0755 (13)	
H2	0.8174	0.0012	0.3912	0.091*	
C6	0.8824 (6)	0.3245 (5)	0.3419 (5)	0.0719 (12)	
H6	0.8352	0.4019	0.3690	0.086*	
C1	0.8022 (5)	0.1976 (5)	0.3980 (4)	0.0578 (10)	
C10	0.6433 (6)	0.1801 (6)	0.5068 (4)	0.0692 (12)	
C11	0.4077 (14)	0.311 (2)	0.6100 (10)	0.079 (4)	0.76 (4)
H11B	0.4195	0.3899	0.6433	0.095*	0.76 (4)
H11A	0.4065	0.2247	0.6891	0.095*	0.76 (4)
C12	0.2393 (16)	0.3312 (14)	0.5389 (12)	0.083 (3)	0.76 (4)
H12A	0.2379	0.4206	0.4660	0.125*	0.76 (4)
H12B	0.1366	0.3295	0.6055	0.125*	0.76 (4)
H12C	0.2349	0.2565	0.4994	0.125*	0.76 (4)
C11A	0.397 (4)	0.233 (6)	0.602 (4)	0.093 (11)	0.24 (4)
H11C	0.3381	0.1699	0.5672	0.111*	0.24 (4)
H11D	0.4249	0.1857	0.6974	0.111*	0.24 (4)
C12A	0.301 (7)	0.370 (6)	0.581 (6)	0.126 (17)	0.24 (4)
H12D	0.3747	0.4323	0.6029	0.188*	0.24 (4)
H12E	0.1907	0.3594	0.6419	0.188*	0.24 (4)
H12F	0.2732	0.4094	0.4860	0.188*	0.24 (4)

### Atomic displacement parameters (Å^2^)

**Table:**

	*U*^11^	*U*^22^	*U*^33^	*U*^12^	*U*^13^	*U*^23^
I1	0.05640 (17)	0.05706 (18)	0.0804 (2)	−0.01560 (12)	0.00974 (13)	−0.02719 (14)
N1	0.0453 (16)	0.0458 (17)	0.0531 (18)	−0.0050 (13)	0.0003 (14)	−0.0144 (14)
O1	0.0593 (18)	0.123 (3)	0.067 (2)	0.0124 (19)	0.0189 (16)	−0.0077 (19)
O2	0.101 (3)	0.109 (3)	0.083 (2)	−0.041 (2)	0.025 (2)	−0.007 (2)
C7	0.076 (3)	0.058 (3)	0.094 (3)	−0.010 (2)	0.024 (3)	−0.038 (2)
C9	0.069 (3)	0.054 (2)	0.056 (2)	−0.0056 (19)	0.005 (2)	−0.0074 (18)
C8	0.044 (2)	0.101 (3)	0.063 (3)	−0.017 (2)	−0.0006 (19)	−0.023 (2)
C4	0.0404 (18)	0.044 (2)	0.052 (2)	−0.0057 (15)	0.0008 (16)	−0.0140 (16)
C3	0.078 (3)	0.055 (3)	0.076 (3)	−0.021 (2)	0.011 (2)	−0.027 (2)
C5	0.053 (2)	0.050 (2)	0.084 (3)	−0.0090 (18)	0.018 (2)	−0.013 (2)
C2	0.076 (3)	0.070 (3)	0.075 (3)	−0.037 (2)	0.020 (2)	−0.017 (2)
C6	0.055 (2)	0.061 (3)	0.089 (3)	−0.003 (2)	0.019 (2)	−0.019 (2)
C1	0.048 (2)	0.066 (3)	0.055 (2)	−0.0092 (18)	−0.0023 (18)	−0.014 (2)
C10	0.057 (2)	0.101 (4)	0.045 (2)	−0.025 (3)	−0.002 (2)	−0.012 (2)
C11	0.055 (5)	0.118 (11)	0.058 (4)	−0.005 (5)	0.018 (4)	−0.026 (5)
C12	0.062 (6)	0.105 (7)	0.078 (6)	−0.004 (4)	0.015 (4)	−0.031 (4)
C11A	0.078 (17)	0.12 (3)	0.087 (17)	−0.043 (18)	0.029 (13)	−0.046 (18)
C12A	0.08 (3)	0.19 (4)	0.12 (3)	−0.02 (2)	0.06 (3)	−0.08 (3)

### Geometric parameters (Å, °)

**Table:**

N1—C4	1.501 (5)	C3—H3	0.9300
N1—C7	1.503 (5)	C5—C6	1.377 (5)
N1—C9	1.510 (5)	C5—H5	0.9300
N1—C8	1.516 (5)	C2—C1	1.356 (6)
O1—C10	1.312 (6)	C2—H2	0.9300
O1—C11	1.483 (9)	C6—C1	1.376 (6)
O1—C11A	1.54 (3)	C6—H6	0.9300
O2—C10	1.204 (5)	C1—C10	1.507 (6)
C7—H7A	0.9600	C11—C12	1.49 (2)
C7—H7B	0.9600	C11—H11B	0.9700
C7—H7C	0.9600	C11—H11A	0.9700
C9—H9A	0.9600	C12—H12A	0.9600
C9—H9B	0.9600	C12—H12B	0.9600
C9—H9C	0.9600	C12—H12C	0.9600
C8—H8A	0.9600	C11A—C12A	1.46 (8)
C8—H8B	0.9600	C11A—H11C	0.9700
C8—H8C	0.9600	C11A—H11D	0.9700
C4—C5	1.370 (5)	C12A—H12D	0.9600
C4—C3	1.374 (5)	C12A—H12E	0.9600
C3—C2	1.397 (6)	C12A—H12F	0.9600
			
C4—N1—C7	111.8 (3)	C6—C5—C4	119.9 (4)
C4—N1—C9	111.2 (3)	C6—C5—H5	120.0
C7—N1—C9	107.3 (3)	C4—C5—H5	120.0
C4—N1—C8	108.3 (3)	C1—C2—C3	122.0 (4)
C7—N1—C8	109.4 (3)	C1—C2—H2	119.0
C9—N1—C8	108.8 (3)	C3—C2—H2	119.0
C10—O1—C11	121.3 (8)	C5—C6—C1	121.1 (4)
C10—O1—C11A	94 (2)	C5—C6—H6	119.5
C11—O1—C11A	31.7 (16)	C1—C6—H6	119.5
N1—C7—H7A	109.5	C2—C1—C6	118.3 (4)
N1—C7—H7B	109.5	C2—C1—C10	120.5 (4)
H7A—C7—H7B	109.5	C6—C1—C10	121.2 (4)
N1—C7—H7C	109.5	O2—C10—O1	125.2 (5)
H7A—C7—H7C	109.5	O2—C10—C1	122.7 (5)
H7B—C7—H7C	109.5	O1—C10—C1	112.1 (4)
N1—C9—H9A	109.5	O1—C11—C12	106.3 (9)
N1—C9—H9B	109.5	O1—C11—H11B	110.5
H9A—C9—H9B	109.5	C12—C11—H11B	110.5
N1—C9—H9C	109.5	O1—C11—H11A	110.5
H9A—C9—H9C	109.5	C12—C11—H11A	110.5
H9B—C9—H9C	109.5	H11B—C11—H11A	108.7
N1—C8—H8A	109.5	C12A—C11A—O1	91 (4)
N1—C8—H8B	109.5	C12A—C11A—H11C	113.5
H8A—C8—H8B	109.5	O1—C11A—H11C	113.5
N1—C8—H8C	109.5	C12A—C11A—H11D	113.5
H8A—C8—H8C	109.5	O1—C11A—H11D	113.5
H8B—C8—H8C	109.5	H11C—C11A—H11D	110.8
C5—C4—C3	120.3 (4)	C11A—C12A—H12D	109.5
C5—C4—N1	118.1 (3)	C11A—C12A—H12E	109.5
C3—C4—N1	121.7 (3)	H12D—C12A—H12E	109.5
C4—C3—C2	118.4 (4)	C11A—C12A—H12F	109.5
C4—C3—H3	120.8	H12D—C12A—H12F	109.5
C2—C3—H3	120.8	H12E—C12A—H12F	109.5
			
C7—N1—C4—C5	−170.5 (4)	C5—C6—C1—C2	−1.5 (7)
C9—N1—C4—C5	−50.5 (5)	C5—C6—C1—C10	177.6 (4)
C8—N1—C4—C5	69.0 (4)	C11—O1—C10—O2	5.2 (10)
C7—N1—C4—C3	11.5 (5)	C11A—O1—C10—O2	−11.3 (17)
C9—N1—C4—C3	131.4 (4)	C11—O1—C10—C1	−174.8 (8)
C8—N1—C4—C3	−109.1 (4)	C11A—O1—C10—C1	168.7 (16)
C5—C4—C3—C2	−2.1 (7)	C2—C1—C10—O2	20.7 (7)
N1—C4—C3—C2	175.9 (4)	C6—C1—C10—O2	−158.4 (5)
C3—C4—C5—C6	1.1 (7)	C2—C1—C10—O1	−159.3 (4)
N1—C4—C5—C6	−177.0 (4)	C6—C1—C10—O1	21.6 (6)
C4—C3—C2—C1	1.3 (7)	C10—O1—C11—C12	−104.2 (13)
C4—C5—C6—C1	0.7 (7)	C11A—O1—C11—C12	−72 (4)
C3—C2—C1—C6	0.5 (7)	C10—O1—C11A—C12A	−174 (4)
C3—C2—C1—C10	−178.7 (4)	C11—O1—C11A—C12A	34 (5)

### Hydrogen-bond geometry (Å, °)

**Table:**

*D*—H···*A*	*D*—H	H···*A*	*D*···*A*	*D*—H···*A*
C5—H5···I1^i^	0.93	3.02	3.932 (4)	166.
C11—H11A···O2	0.97	2.46	2.792 (18)	100.

Symmetry codes: (i) *x*+1, *y*, *z*.
